# Dual-luminescent Sc_2_(MoO_4_)_3_:Dy^3+^/Eu^3+^ phosphor system: energy transfer dynamics and high-sensitivity temperature sensing

**DOI:** 10.1039/d5ra04860e

**Published:** 2025-08-15

**Authors:** Zhengrong Xia, Rongqing Li, Fangfang Liu, Weiwei Zhou, Wang Zhao, Wei Meng, Mingjun Song, Junpeng Xue

**Affiliations:** a Anhui Engineering Research Center for Photoelectrocatalytic Electrode Materials, School of Electrical Engineering, Huainan Normal University Huainan 232038 China wwzhou@hnnu.edu.cn; b School of Science, Jiangsu University of Science and Technology Zhenjiang 212100 China xjplane@126.com; c School of Chemistry, Chemical and Environmental Engineering, Weifang University Weifang 261061 China smj521209@126.com

## Abstract

A series of Dy^3+^/Eu^3+^ co-doped Sc_2_(MoO_4_)_3_ (SMO) phosphors were synthesized *via* a conventional solid-state reaction. Phase purity and morphology were characterized by X-ray diffraction with Rietveld refinement and scanning electron microscopy, confirming the formation of phase-pure orthorhombic crystals. The electronic structure was investigated through density functional theory calculations combined with diffuse reflectance spectroscopy. Under 266 and 352 nm excitation, the Dy^3+^ doped SMO phosphors exhibited characteristic emission peaks at 488 nm (^4^F_9/2_ → ^6^H_15/2_), 578 nm (^4^F_9/2_ → ^6^H_13/2_), 667 nm (^4^F_9/2_ → ^6^H_11/2_), and 761 nm (^4^F_9/2_ → ^6^H_9/2_). In the Dy^3+^/Eu^3+^ co-doped systems, detailed analysis of spectra and luminescence decay kinetics quantitatively confirmed efficient energy transfer from Dy^3+^ to Eu^3+^. Finally, the optimized SMO:Dy^3+^, Eu^3+^ phosphors achieved maximum relative (*S*_r_) and absolute (*S*_a_) sensitivities of 3.817% K^−1^ and 0.018 K^−1^, respectively, demonstrating potential for ratiometric optical thermometry applications.

## Abstract 2

Energy transfer from Dy^3+^ to Eu^3+^ was observed in the Sc_2_(MoO_4_)_3_ host and the distinct temperature-dependent luminescence behaviors of these ions were exploited for potential temperature sensing applications.

## Graphical abstract

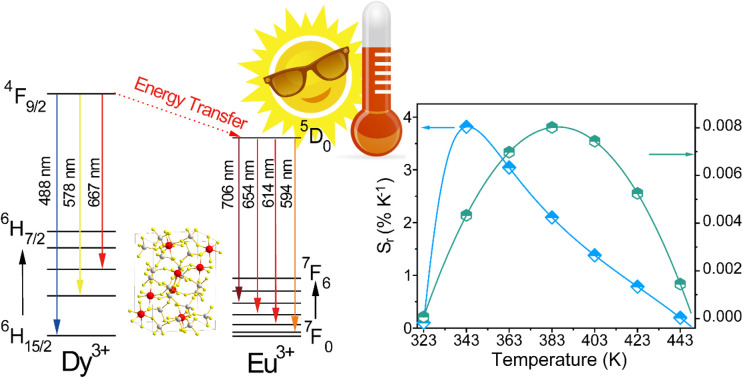

## 1. Introduction

High-precision temperature sensing technology plays a vital role in scientific research, industrial monitoring, and biomedical applications, where accurate thermal characterization is crucial for material studies and system regulation.^1,2^ However, conventional contact-based sensors exhibit significant limitations: they are susceptible to electromagnetic interference, suffer from thermal conduction delays, and have restricted spatial resolution, making precise and noninvasive measurements challenging in extreme environments (*e.g.*, high-temperature reactors, high-speed fluid flows) or living tissues.^3^ To address these challenges, optical thermometry has emerged as an innovative solution. This technique establishes a quantitative correlation between the temperature of luminescent materials and their optical parameters, such as fluorescence intensity ratio (FIR), lifetime, and spectral shift, enabling noncontact measurements with advantages including immunity to electromagnetic interference, rapid response time (microsecond scale), and high spatial resolution (micrometer scale).^4^ Among these methods, the dual-emission centers FIR technique represents a major breakthrough. By utilizing two emission centers with distinct thermal quenching behaviors as the reference and detection signals, this approach not only compensates for excitation source fluctuations and optical path losses but also overcomes the inherent limitations of traditional rare-earth-ion-based thermally coupled levels (TCLs), where the narrow energy gap (200–2000 cm^−1^) leads to low relative sensitivity and poor signal discriminability.^5,6^ This innovative strategy significantly enhances measurement reliability while achieving higher sensitivity and a broader operational temperature range, demonstrating great potential for diverse applications.

In recent years, various ion pairs including Mn^2+^/Mn^4+^, Bi^3+^/Eu^3+^, Er^3+^/Yb^3+^, Eu^2+^/Eu^3+^, Dy^3+^/Eu^3+^, Dy^3+^/Mn^4+^, have demonstrated excellent temperature-sensing performance as dual-emission centers in optical thermometry.^1,7–10^ Among these, the Dy^3+^/Eu^3+^ pair has attracted particular attention due to its distinctive temperature-responsive characteristics. Under UV or blue light excitation, Dy^3+^ exhibits remarkable temperature sensitivity in the FIR between its 575 nm (^4^F_9/2_ → ^4^H_13/2_) and 480 nm (^4^F_9/2_ → ^4^H_15/2_) emissions, while Eu^3+^ shows significant temperature-dependent lifetime variations at about 615 nm (^5^D_0_ → ^7^F_2_).^11,12^ Notably, Dy^3+^ demonstrates negative thermal quenching behavior in specific host matrices, which substantially extends the material's applicability in high-temperature regimes.^13,14^ More importantly, the energy transfer between Dy^3+^ and Eu^3+^ can reverse Eu^3+^'s thermal quenching behavior from “positive” to “negative”, leading to synergistic enhancement of luminescence efficiency at elevated temperatures.^15^ This unique interaction not only enables highly sensitive dual-mode FIR thermometry over a broad temperature range (300–500 K), but also allows tunable emission colors from cool white to warm red through controlled doping ratios, providing an important foundation for developing multifunctional optical devices.

The selection of host materials is crucial for optimizing the luminescent performance of rare-earth ions. In the field of optical temperature sensing, A_2_M_3_O_12_-type materials (where A represents trivalent rare-earth ions and M denotes W^6+^ or Mo^6+^) exhibiting negative thermal expansion (NTE) behavior have attracted significant attention.^16–19^ As well known, these materials demonstrate unique lattice contraction upon heating, which reduces the interatomic distances between activator ions, enhances energy transfer efficiency, effectively suppresses thermal quenching, and may even induce thermally enhanced luminescence. For instance, NTE materials such as Sc_2_W_3_O_12_:Eu^3+^, CaMoO_4_:Sm^3+^, Sc_2_W_3_O_12_:Dy^3+^ and Yb_2_W_3_O_12_:Er^3+^ exhibit outstanding luminescent performance even at elevated temperatures.^20–23^ Among them, SMO stands out as a representative NTE material with remarkable thermal stability and chemical durability, ensuring reliable performance under harsh conditions. Notably, SMO: Eu^3+^ maintains over 90% of its initial luminescence intensity even at 300 °C, demonstrating exceptional potential for high-temperature applications.^24^ In this study, Dy^3+^/Eu^3+^ co-doped SMO phosphors were synthesized *via* solid-state reaction. The structural properties, bandgap characteristics, and luminescent behavior were systematically investigated through X-ray diffraction (XRD), scanning electron microscopy (SEM), diffuse reflectance spectroscopy (DRS), photoluminescence (PL) spectroscopy, and density functional theory (DFT) calculations. Furthermore, the distinct thermal responses of Dy^3+^ and Eu^3+^ ions were exploited to demonstrate their potential for non-contact optical thermometry.

## 2. Results and discussion

### 2.1 Crystalline structure and morphology

**Fig. 1:**
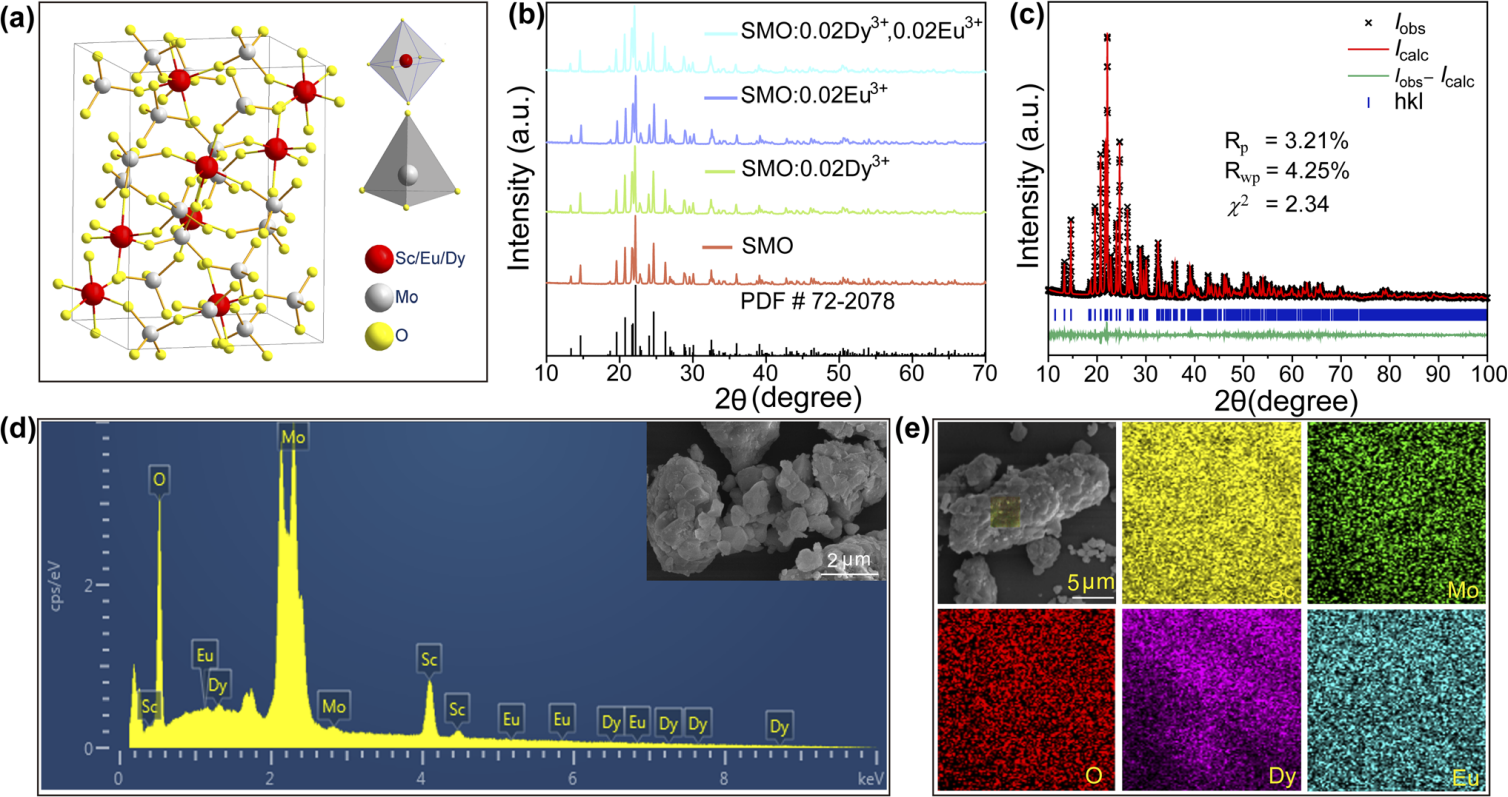
(a) The crystal structure of SMO:Dy^3+^,Eu^3+^ with the ScO_6_ and MoO_4_ polyhedrons; (b) XRD patterns of SMO, SMO:0.02Dy^3+^, SMO:0.02Eu^3+^ and SMO:0.02Dy^3+^,0.02Eu^3+^ phosphors; (c) Rietveld refinement of SMO:0.02Dy^3+^,0.02Eu^3+^ phosphors; (d) the EDS spectrum, SEM image and (e) elemental mapping images of SMO:0.02Dy^3+^,0.02Eu^3+^ phosphor.

Fig. 1a shows the crystal structure of SMO: 0.02Dy^3+^,0.02Eu^3+^, which crystallizes in the orthorhombic system with space group *Pbcn*. The lattice parameters of SMO are determined to be *a* = 13.242 Å, *b* = 9.544 Å, *c* = 9.637 Å, and the unit cell volume *V* = 1217.49 Å^3^. In this structure, Sc^3+^ ions coordinate with six oxygen atoms to form ScO_6_ octahedra, while Mo^6+^ ions coordinate with four oxygen atoms to form MoO_4_ tetrahedra. These ScO_6_ octahedra and MoO_4_ tetrahedra are interconnected through corner-sharing oxygen atoms, constructing a stable three-dimensional Sc–O–Mo framework. In assessing Dy^3+^ and Eu^3+^ ion substitution within the crystal structure, the difference in ionic radius (*D*_r_) was determined using the equation:1
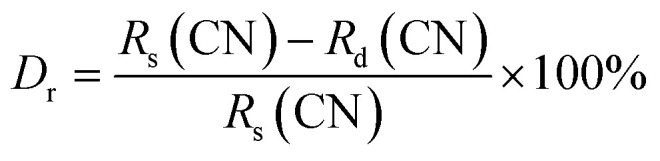
where *D*_r_ signifies the percentage variation in radius, CN indicates the coordination number, and *R*_s_ and *R*_d_ refer to the radii of the central and substituted ions, respectively. When Dy^3+^ (*r* = 0.91 Å, CN = 6) and Eu^3+^ (*r* = 0.95 Å, CN = 6) ions are doped into SMO, they may potentially occupy Sc^3+^ sites (*r* = 0.75 Å, CN = 6) or Mo^6+^ sites (*r* = 0.41 Å, CN = 4). The calculated ionic radius variation for Dy^3+^ indicates that *D*_r(Sc–Dy)_ is 21.33%, while *D*_r(Mo–Dy)_ is 121.9%. Taking into account the similarity in ionic radii and charge balance considerations, Dy^3+^ ions preferentially occupy Sc^3+^ sites rather than Mo^6+^ sites. Similarly, the Eu^3+^ ions in the SMO: Dy^3+^,Eu^3+^ sample also exhibit a preference for occupying Sc^3+^ sites. This selective substitution primarily arises from the close match in ionic radii and coordination environment between the dopant ions and Sc^3+^, while effectively maintaining the structural stability of the host lattice. Fig. 1b presents the XRD patterns of the SMO host, SMO:0.02Dy^3+^, SMO:0.02Eu^3+^, and SMO:0.02Dy^3+^,0.02Eu^3+^ phosphors, respectively. As shown, all the diffraction peaks of the synthesized samples exhibit similar profiles and match well with the standard card of SMO (PDF #72-2078), confirming the successful preparation of phase-pure phosphors without any impurities. This result further demonstrates that the incorporation of Dy^3+^ and Eu^3+^ ions into the host lattice does not affect the phase purity. To further analyze the crystal structure of the phosphors, Rietveld refinement of the lattice parameters for SMO: 0.02Dy^3+^,0.02Eu^3+^ was performed using GSAS software, as shown in Fig. 1c. The refinement yielded reliable factors of *R*_wp_ = 4.25%, *R*_p_ = 3.21%, and *χ*^2^ = 2.34, confirming the formation of a pure orthorhombic phase without any secondary phases. The refined unit cell parameters are *a* = 13.284 Å, *b* = 9.554 Å, *c* = 9.642 Å, and *V* = 1223.66 Å^3^. Notably, the expansion of the lattice parameters provides clear evidence that Dy^3+^ and Eu^3+^ ions have been successfully incorporated into the SMO lattice. The SMO: 0.02Dy^3+^, 0.02Eu^3+^ phosphor was selected as a representative to investigate the morphology of the synthesized phosphors. As shown in the inset of Fig. 1d and S1, the SEM images reveal that the prepared phosphors exhibit irregular morphology with particle sizes of micrometer level. Furthermore, SEM mapping was performed to examine the elemental distribution in the synthesized sample (Fig. 1d), demonstrating the overall elemental dispersion. Fig. 1e displays the specific distributions of Sc, Mo, O, Dy, and Eu, respectively, showing homogeneous distribution of these elements across the scanned area, which confirms the successful incorporation of Dy^3+^/Eu^3+^ ions.

### 2.2 Electronic band structure and diffuse reflection spectra

**Fig. 2:**
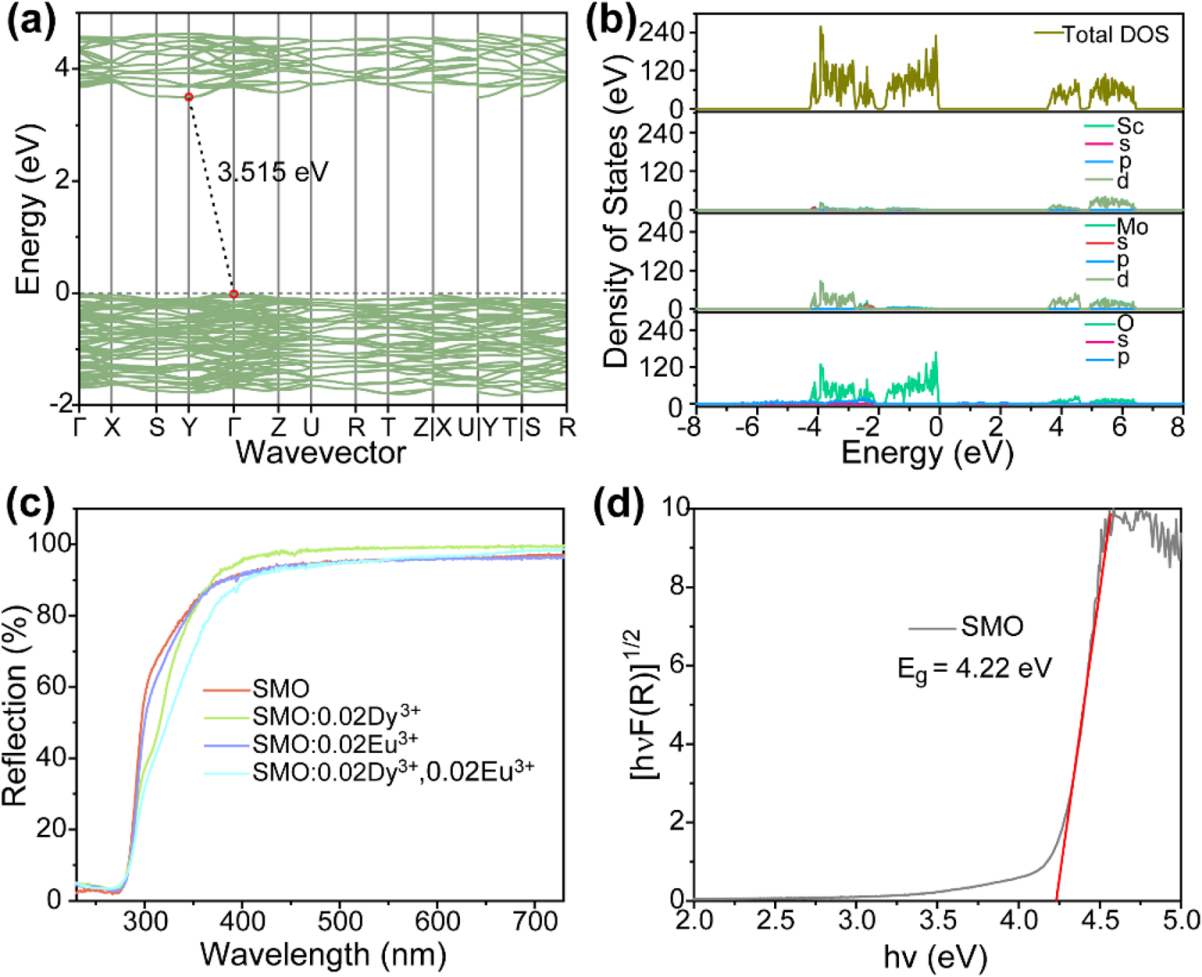
(a) Calculated electronic band structure of SMO; (b) DOS of SMO; (c) DRS of the SMO, SMO: 0.02Dy^3+^, SMO:0.02Eu^3+^ and SMO:0.02Dy^3+^,0.02Eu^3+^ phosphors; (d) the band gap of pure SMO.

To elucidate the electronic structure characteristics of SMO, the DFT calculations on its electronic properties were performed in detail (see SI). As shown in Fig. 2a, the theoretical calculations reveal that SMO exhibits an indirect bandgap of 3.515 eV, with the valence band maximum (VBM) located at the *Γ* point and the conduction band minimum (CBM) at the *Y* point in the Brillouin zone. These results unambiguously confirm the indirect bandgap semiconductor nature of this material. Furthermore, its wide bandgap characteristic (>3.3 eV) is crucial for facilitating effective electronic transitions in rare-earth luminescent centers. To further investigate the electronic band structure, the density of states (DOS) and partial density of states (PDOS) were calculated. As shown in Fig. 2b, the valence band (−4.3 to 0 eV) is primarily contributed by O and Mo atoms, while the conduction band mainly originates from Sc and Mo atoms, particularly their d-orbitals. In addition, the DRS was performed on representative samples and the corresponding bandgap values were calculated. As shown in Fig. 2c, all samples exhibit a significant reflectance drop around 270 nm, which corresponds to the absorption band of SMO. Upon doping Dy^3+^ and Eu^3+^ ions into the SMO host, several weak absorption peaks are observed in the 300–600 nm range. These features can be attributed to the 4f–4f transitions of Dy^3+^ and Eu^3+^ ions. The optical band gap (*E*_g_) of the material was based on the method proposed by Wood and Tauc, as follows:^25^

2 *αhv* = *A*(*hv* − *E*_g_)^*n*/2^

where *α* is the absorption coefficient, *hν* represents the incident photon energy, *E*_g_ denotes the optical band gap, and *A* is a proportionality constant. The exponent *n* characterizes the nature of the electronic transitions, with *n* = 1 for direct band gap semiconductors and *n* = 4 for indirect band gap materials. By fitting the experimental data (Fig. 2d) with this equation, the optical band gap of SMO was determined to be approximately 4.22 eV. This experimentally obtained value is slightly larger than that calculated using DFT, which can be attributed to the well-known band gap underestimation caused by the local density approximation (LDA) method employed in DFT calculations.^26^

### 2.3 Photoluminescence properties and energy transfer mechanism

**Fig. 3:**
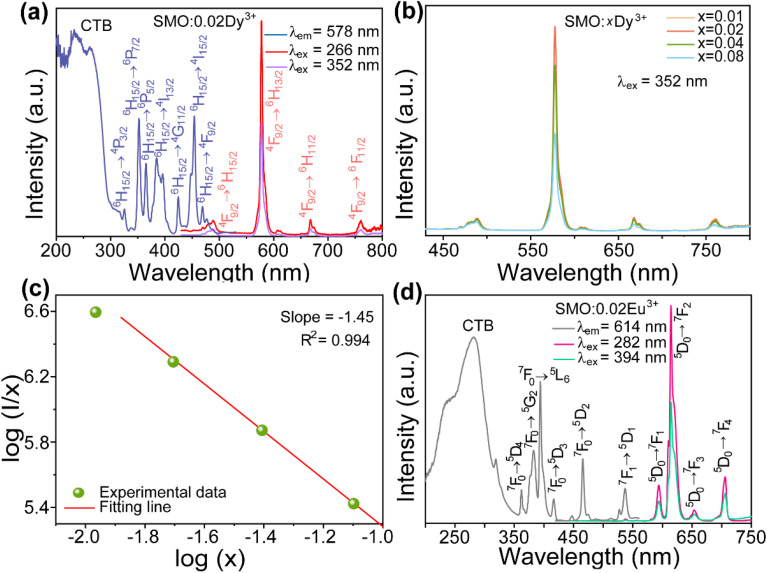
(a) PLE and PL spectra of SMO:0.02Dy^3+^ phosphor; (b) PL spectra of SMO:*x*Dy^3+^ phosphors; (c) the linear fitting of log(*I*/*x*) *vs.* log(*x*); (d) PLE and PL spectrum of SMO:0.02Eu^3+^ phosphor.

Building upon the excellent host properties of SMO, we selected Dy^3+^ and Eu^3+^ as dopants for detailed luminescence studies. Fig. 3a presents the excitation and emission spectra of SMO: 0.02Dy^3+^ phosphors. As shown, the excitation spectrum monitored at 578 nm (corresponding to the ^4^F_9/2_ → ^6^H_13/2_ transition) exhibit a broad charge transfer band (CTB) centered at 266 nm, attributed to the overlap of O^2−^ → Mo^6+^ LMCT and O^2−^ → Dy^3+^ CTB.^24,27^ Additionally, several sharp peaks between 300-500 nm are observed, corresponding to characteristic f–f transitions of Dy^3+^, namely ^6^H_15/2_ → ^4^P_3/2_ (325 nm), ^6^H_15/2_ → ^6^P_7/2_ (352 nm), ^6^H_15/2_ → ^6^P_5/2_ (365 nm), ^6^H_15/2_ → ^4^I_13/2_ (384 nm), ^6^H_15/2_ → ^4^G_11/2_ (424 nm), ^6^H_15/2_ → ^4^I_15/2_ (454 nm) and ^6^H_15/2_ → ^4^F_9/2_ (469 nm), respectively.^27^Fig. 3a also presents the PL spectrum of SMO: 0.02Dy^3+^ under 266 and 352 nm excitation, exhibiting four characteristic emission peaks at 488 nm (^4^F_9/2_ → ^6^H_15/2_), 578 nm (^4^F_9/2_ → ^6^H_13/2_), 667 nm (^4^F_9/2_ → ^6^H_11/2_), and 761 nm (^4^F_9/2_ → ^6^F_11/2_), corresponding to the characteristic 4f–4f transitions of Dy^3+^.^28^ The most intense emission at 578 nm (yellow region) significantly surpasses the 488 nm (blue) emission intensity, which is attributed to their distinct transition origins: the 488 nm emission arises from a magnetic dipole (MD) transition (^4^F_9/2_ → ^6^H_15/2_), while the 578 nm emission originates from an electric dipole (ED) transition (^4^F_9/2_ → ^6^H_13/2_).^23^ The dominant ED transition at 578 nm not only indicates its hypersensitive nature to the local crystal field environment but also suggests a strong influence of the host lattice on luminescence behavior of Dy^3+^. Fig. 3b displays the emission spectra of SMO: *x*Dy^3+^ phosphors (*x* = 0.01, 0.02, 0.04, and 0.08) under 352 nm excitation. All samples exhibit similar spectral profiles but varying emission intensities. The luminescence intensity initially increases with Dy^3+^ concentration, reaching a maximum at 2 mol% doping, beyond which concentration quenching occurs. This phenomenon can be explained by the enhanced non-radiative energy transfer (ET) between neighboring Dy^3+^ ions at higher concentrations, which becomes the dominant decay pathway when the critical distance between activators is exceeded, as follows:^29,30^3
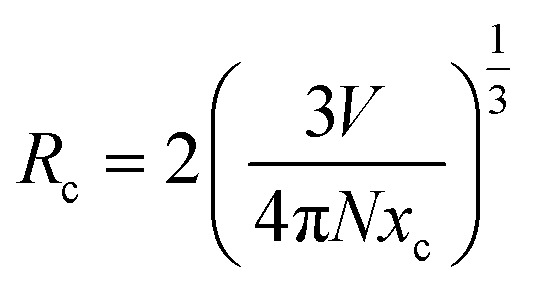
here, *R*_c_, *V*, *x*_c_, and *N* denote the critical distance, unit cell volume (1223.66 Å^3^ for SMO), optimal Dy^3+^ doping concentration (0.02), and number of cation sites per unit cell (8), respectively. The calculated *R*_c_ value of 24.5 Å for this phosphor system significantly exceeds the 5 Å threshold for exchange interaction dominance in rare-earth ions. This quantitative analysis confirms that multipolar interactions constitute the primary mechanism for concentration quenching in this material system. The non-radiative energy transfer mechanism in SMO:*x*Dy^3+^ phosphors was further elucidated using Dexter's theoretical formula:^31^4
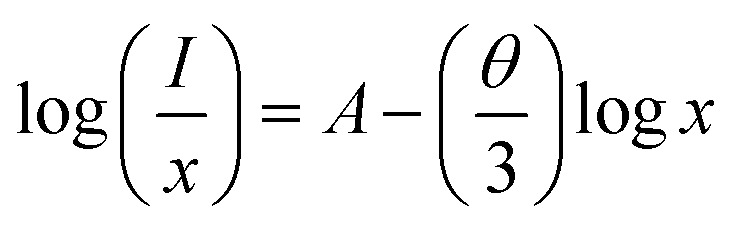
where *x*, *I* and *A* represent the activator concentration, emission intensity, and the constant, respectively. *θ* denotes the interaction type between rare-earth ions: *θ* = 6 for electric dipole–dipole interaction, *θ* = 8 for dipole–quadrupole interaction, and *θ* = 10 for quadrupole–quadrupole interaction. Fitting the experimental data of emission intensity *versus* Dy^3+^ concentration (solid curve in Fig. 3c) yielded a *θ* value of 4.35, being close to 6, clearly demonstrates that the concentration quenching of Dy^3+^ in the SMO host primarily occurs through electric dipole–dipole interactions. Fig. 3d presents the PLE spectrum of SMO: 0.02Eu^3+^ phosphor monitored at the characteristic emission wavelength of 614 nm. The spectrum exhibits two distinct features, namely a broad asymmetric CTB spanning 200–350 nm, primarily attributed to the overlapping charge transfer transitions of O^2−^–Eu^3+^ and O^2−^–Mo^6+^ and several sharp peaks corresponding to the 4f–4f transitions of Eu^3+^ ions. The narrow excitation peaks observed at 362 nm (^7^F_0_ → ^5^D_4_), 382 nm (^7^F_0_ → ^5^G_2_), 394 nm (^7^F_0_ → ^5^L_6_), 416 nm (^7^F_0_ → ^5^D_3_), and 465 nm (^7^F_0_ → ^5^D_2_) represent ground state absorption transitions, while those at 537 nm (^7^F_1_ → ^5^D_1_) and 593 nm (^7^F_1_ → ^5^D_0_) originate from excited state absorption processes.^32^ Notably, the excitation spectrum reveals two dominant peaks at 280 nm (CTB) and 394 nm (^7^F_0_ → ^5^L_6_ transition), with the former exhibiting the highest intensity. Under 282 nm and 394 nm excitation, the emission spectrum of SMO:0.02Eu^3+^ exhibits characteristic peaks at 538 nm (^5^D_1_ → ^7^F_0_), 594 nm (^5^D_0_ → ^7^F_1_), 614 nm (^5^D_0_ → ^7^F_2_), 654 nm (^5^D_0_ → ^7^F_3_), and 706 nm (^5^D_0_ → ^7^F_4_), as shown in Fig. 3d.^32^ The dominant emission at 614 nm corresponds to the hypersensitive electric dipole transition, whose intensity is strongly influenced by the local crystal field symmetry. In contrast, the magnetic dipole transition at 594 nm remains relatively insensitive to the surrounding environment. The intensity ratio *R* (*I*_614_/*I*_594_) of 5.98 for SMO: 0.02Eu^3+^ clearly indicates that Eu^3+^ ions occupy low-symmetry sites lacking inversion centers, consistent with our observations for Dy^3+^-doped sample (see Fig. 3a).

**Fig. 4:**
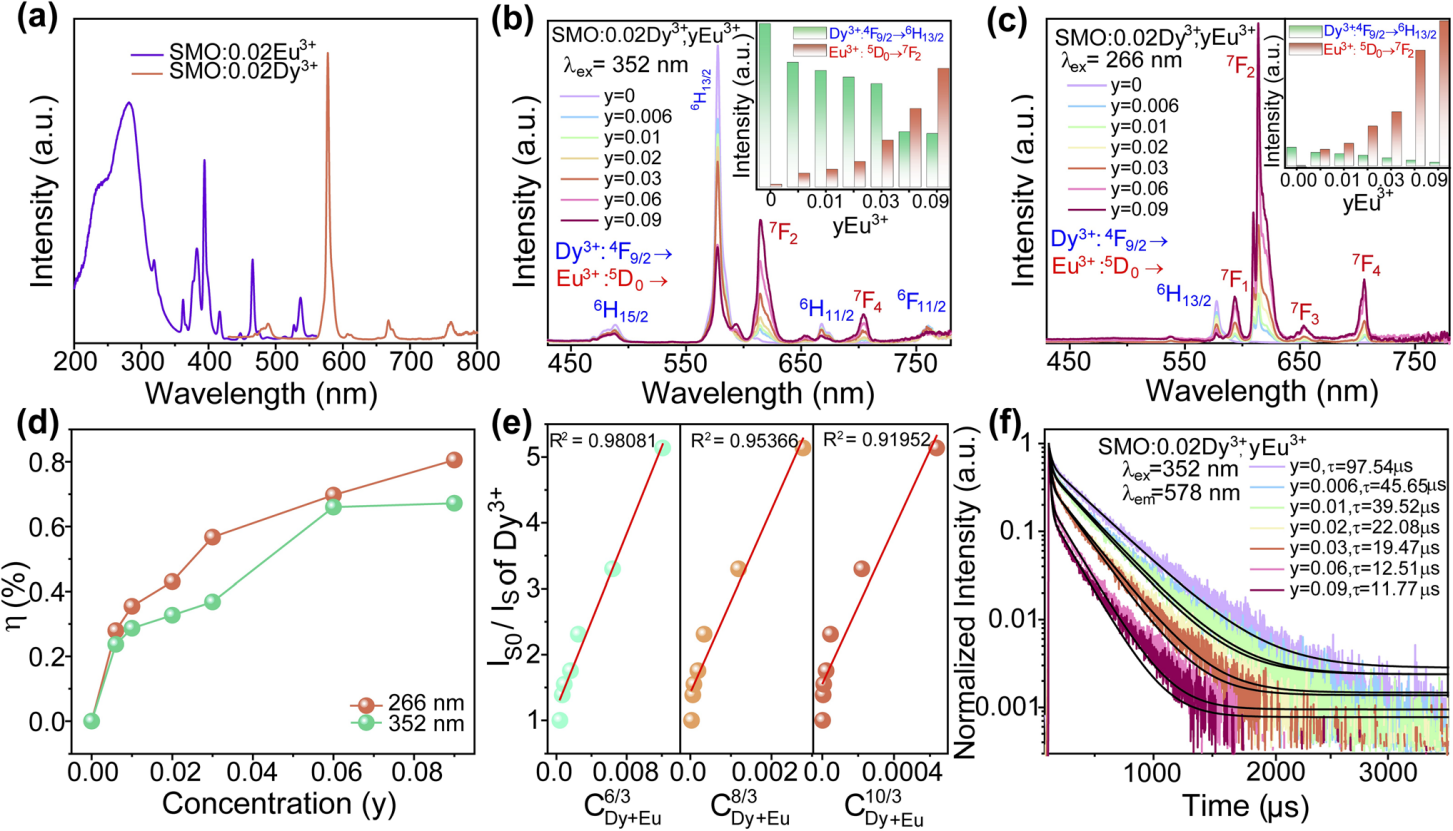
(a) PLE spectra of SMO:0.02Eu^3+^ and PL spectra of the SMO:0.02Dy^3+^ phosphors; PL spectrum of SMO:0.02Dy^3+^,*y*Eu^3+^ phosphors under (b) 352 and (c) 266 nm excitation; (d) ET efficiency (*η*) of SMO:0.02Dy^3+^,*y*Eu^3+^ phosphors under 352 and 266 nm excitations; (e) Dependence of (*I*_S0_/*I*_S_) of Dy^3+^ on C^6/3^_Dy+Eu_, C^8/3^_Dy+Eu_ and C^10/3^_Dy+Eu_; (f) decay profiles of the SMO:0.02Dy^3+^,*y*Eu^3+^ samples.

Fig. 4a presents the PL spectrum of SMO: 0.02Dy^3+^ and PLE spectrum of SMO: 0.02Eu^3+^ phosphors. Notably, a significant spectral overlap is observed between the emission peaks of Dy^3+^ and the sharp excitation peaks of Eu^3+^, suggesting the possible occurrence of energy transfer (ET) from Dy^3+^ to Eu^3+^. To elucidate the ET mechanism between Dy^3+^ and Eu^3+^ ions, a series of SMO:0.02Dy^3+^,*y*Eu^3+^ samples were successfully synthesized. As shown in Fig. 4b and c, the emission spectrum under 266 and 352 nm excitation exhibit characteristic peaks of both Dy^3+^ and Eu^3+^ ions, whose intensities are strongly dependent on dopant concentration. Notably, with increasing Eu^3+^ content, the emission intensity of Dy^3+^ decreases rapidly while that of Eu^3+^ rises accordingly (see insets of Fig. 4b and c). This opposite variation trend clearly indicates the occurrence of ET from Dy^3+^ to Eu^3+^ ions. The ET efficiency (*η*) was further quantified using the equation:^33^5
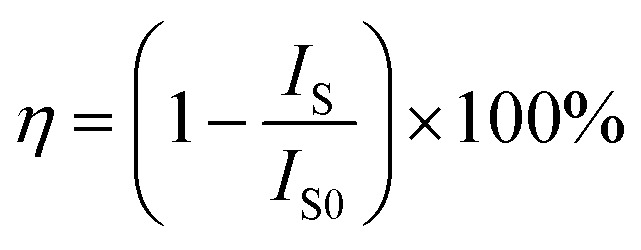
where *I*_S_ and *I*_S0_ represent the emission intensities of Dy^3+^ with and without Eu^3+^ co-doping, respectively. As demonstrated in Fig. 4d, *η* values show a continuous enhancement with increasing Eu^3+^ concentration, reaching maximum efficiencies of 80.53% and 67.2% under 266 and 352 nm excitation. These results confirm the effective ET from Dy^3+^ to Eu^3+^ ions in the SMO host lattice. According to previous reports, the ET mechanism between Dy^3+^ and Eu^3+^ predominantly occurs *via* electric multipolar interactions.^30^ Based on Dexter's theory, the relationship for electric multipolar interactions can be expressed as:^30^6
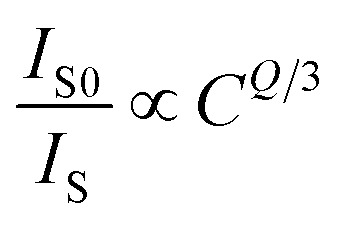
where *I*_S0_ and *I*_S_ represent the emission intensity of SMO:0.02Dy^3+^ without and with Eu^3+^, and *C* is the total concentration of Dy^3+^ and Eu^3+^ ions. As shown in Fig. 4e, the linear correlation between *I*_S0_/*I*_S_ and *C*^*Q*/3^ was established through linear fitting. The optimal fitting parameter *R*^2^ (closest to 1) was achieved at *Q* = 6, confirming that the ET mechanism in SMO:Dy^3+^,Eu^3+^ phosphors originate from dipole–dipole interactions. To corroborate the spectroscopic analysis, the decay curves of SMO:0.02Dy^3+^,*y*Eu^3+^ phosphors monitored at 578 nm emission were investigated (Fig. 4f). The experimental data reveal that the decay curves follow a second-order exponential decay law, expressed mathematically as:^34^7
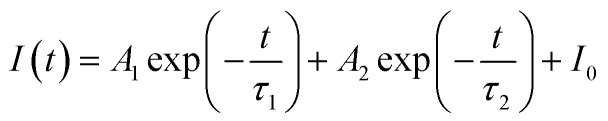
where *I*(*t*) represent the luminescence intensity at time *t*, *A*_1_ and *A*_2_ are constants, and *τ*_1_ and *τ*_2_ correspond to the fast and slow decay components, respectively. Based on this model, the effective decay time (τ*) can be calculated using the following equation:

8 τ* = (*A*_1_*τ*_1_^2^ + *A*_2_*τ*_2_^2^)/(*A*_1_*τ*_1_ + *A*_2_*τ*_2_)

The calculated results demonstrate that for *y* values of 0, 0.006, 0.01, 0.02, 0.03, 0.06, and 0.09, the corresponding decay times are 97.54, 45.65, 39.52, 22.08, 19.47, 12.51, and 11.77 μs, respectively. Notably, the decay time exhibits a monotonic decrease with increasing Eu^3+^ concentration, further providing conclusive experimental evidence for the ET process from Dy^3+^ to Eu^3+^.

### 2.4 Thermal properties

**Fig. 5:**
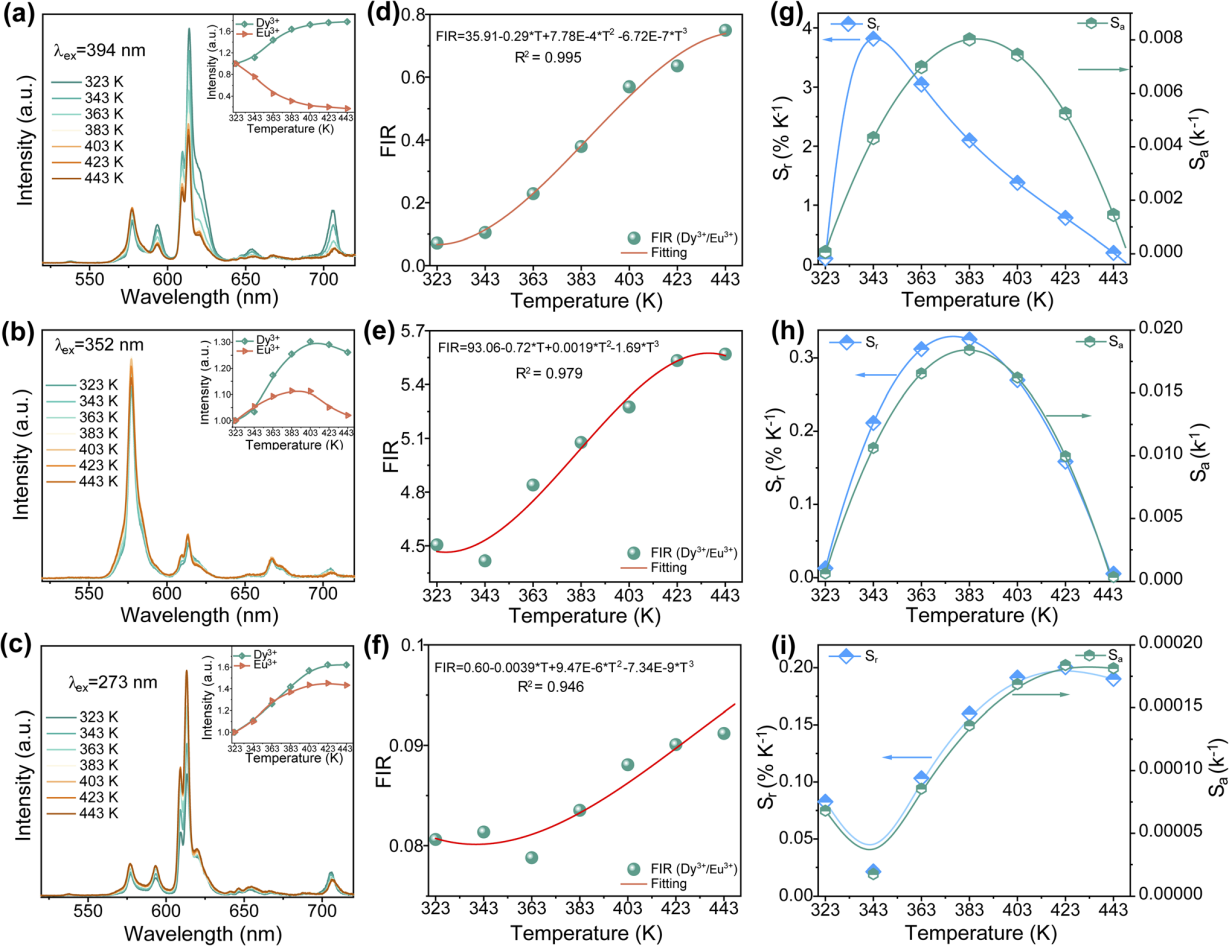
(a–c) Temperature-dependent PL spectra of the SMO: 0.02Dy^3+^,0.02Eu^3+^ sample under 394 nm, 352 nm and 273 nm. (d–f) Temperature-dependent FIR value of Dy^3+^/Eu^3+^ under 394 nm, 352 nm and 273 nm. (g–i) *S*_r_ and *S*_a_ values of SMO: 0.02Dy^3+^,0.02Eu^3+^ phosphor at different temperatures under 394 nm, 352 nm and 273 nm.

To evaluate the potential application of the as-prepared samples in optical thermometry, the PL spectra of SMO:0.02Dy^3+^,0.02Eu^3+^ were measured under different excitation wavelengths (394, 352, and 273 nm) over the temperature range of 323–443 K (Fig. 5a–c). The results reveal distinct temperature-dependent luminescence behaviors under various excitation conditions, namely the emission intensity of Dy^3+^ exhibits a nearly monotonic increase with rising temperature, while the luminescence behavior of Eu^3+^ shows strong excitation-dependent characteristics. Specifically, under 394 nm excitation, the Eu^3+^ emission displays a monotonic decrease due to thermal quenching. In contrast, under 352 and 273 nm excitation, the Eu^3+^ emission demonstrates non-monotonic behavior with initial enhancement followed by attenuation, which likely results from the competition between energy transfer processes and thermal quenching effects. These distinct temperature-dependent luminescent responses between Dy^3+^ and Eu^3+^ ions endow the material with promising optical thermometric properties. As demonstrated in previous studies, the FIR of dual-emitting activators can be calibrated for temperature sensing using two emission signals within a single spectral band, where the FIR is defined as follows:^35^9
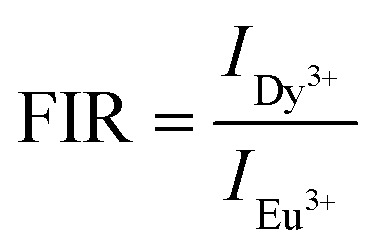


Specifically, within the temperature range of 323–443 K, the FIR values exhibit a monotonic increasing trend with rising temperature, which can be well fitted by polynomial functions (Fig. 5d–f). To quantitatively evaluate the thermometric performance of the material, the absolute sensitivity (*S*_a_) and relative sensitivity (*S*_r_) were employed as key assessment parameters, calculated according to the following equations:^35^10
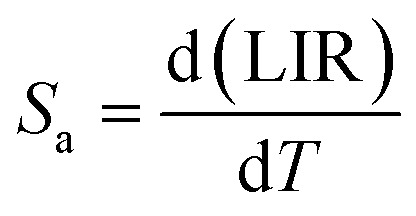
11
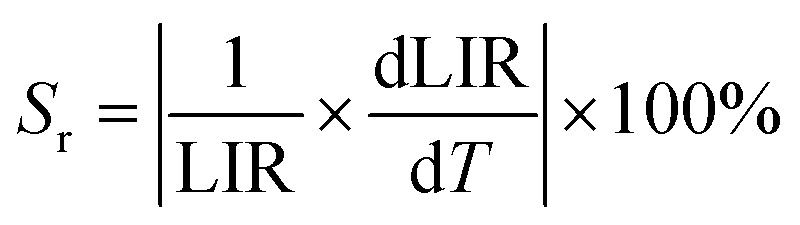


Based on the aforementioned functions and fitted values, the *S*_r_ and *S*_a_ values of SMO:0.02Dy^3+^,0.02Eu^3+^ phosphors were calculated, as shown in Fig. 5g–i. The results demonstrate that within the temperature range of 323–443 K, both *S*_a_ and *S*_r_ values of the samples exhibit distinct variation trends with increasing temperature. Specifically, under excitation wavelengths of 394, 352, and 276 nm, the maximum *S*_r_ values reached 3.817% K^−1^ (at 343 K), 0.33% K^−1^ (at 383 K), and 0.20% K^−1^ (at 423 K), respectively. Meanwhile, the highest *S*_a_ values were 0.0082 K^−1^ (383 K), 0.018 K^−1^ (383 K), and 0.000184 K^−1^ (423 K). Table 1 compares the *S*_r_ and *S*_a_ values of this study with those of other reported temperature sensors. The results demonstrate that among Dy^3+^–Eu^3+^ co-doped temperature sensing materials, our sample exhibits relatively good temperature sensing properties, highlighting its potential for non-contact temperature sensing applications.

**Table 1:** Optical thermometric properties of several typical temperature sensing materials based on the Dy^3+^/Eu^3+^ doped phosphors

Phosphor	Temperature range (K)	*S*_a_ (K^−1^) *T*_max_ (K)	*S*_r_ (% K^−1^) *T*_max_ (K)	Ref.
CaLa_4_Si_3_O_13_:Dy^3+^,Eu^3+^	323–573	—	3.32@323	9
Ca_3_Al_2_Ge_3_O_12_:Dy^3+^,Eu^3+^	303–523	0.000551@303	0.0359@303	11
Ca_2_YNbO_6_:Dy^3+^,Eu^3+^	300–475	0.00469@300	3.14@300	15
Li_2_Ba_5_W_3_O_15_:Dy^3+^,Eu^3+^	303–443	—	0.97@303	28
SrMoO_4_: Dy^3+^,Eu^3+^	300–520	—	1.46@300	32
SrLaGaO_4_:Dy^3+^,Eu^3+^	298–423	0.0061@298	0.36@298	36
BaLa_4_Si_3_O_13_:Dy^3+^,Eu^3+^	298–523	0.205@523	1.462@497	37
NaPbBi_2_(PO_4_)_3_:Dy^3+^,Eu^3+^	303–483	0.0039@463	0.65@443	38
K_2_Ta(PO_4_)_3_:Dy^3+^,Eu^3+^	298–473	0.00153@323	0.16@323	39
KBaGd(WO_4_)_3_:Dy^3+^,Eu^3+^	298–478	0.033@478	0.64@458	40
GdAl_3_(BO_3_)_4_:Dy^3+^,Eu^3+^	300–500	—	1.37@475	41
SrIn_2_(P_2_O_7_)_2_:Dy^3+^,Eu^3+^	298–523	0.00382@293	0.741@293	42
SMO:Dy^3+^,Eu^3+^	323–443	0.0082@383	3.817@343	This work

## 3. Conclusions

In this study, a series of Dy^3+^/Eu^3+^ co-doped SMO phosphors were synthesized *via* conventional solid-state reaction. The electronic band structure was systematically investigated through combined DRS and DFT calculations, with detailed analysis of band structure and DOS providing theoretical insights into the luminescent behavior. Under UV excitation, the phosphors exhibited characteristic emissions originating from Dy^3+^ (^4^F_9/2_ → ^6^H_15/2_, ^6^H_13/2_, ^6^H_11/2_, and ^6^H_9/2_ transitions) and Eu^3+^ (^5^D_0_ → ^7^F_*J*_, *J* = 0–4 transitions). Efficient ET from Dy^3+^ to Eu^3+^ was achieved through dipole–dipole interaction, enabling tunable multicolor emission. Temperature-dependent photoluminescence studies (323–423 K) on SMO:0.02Dy^3+^,0.02Eu^3+^ revealed distinct thermal responses between the two activators, yielding maximum *S*_a_ and *S*_r_ values of 0.018 K^−1^ and 3.817% K^−1^, respectively. These findings demonstrate the potential of Dy^3+^/Eu^3+^ co-doped SMO phosphors as high-performance optical thermometric materials for non-contact temperature sensing applications.

## Conflicts of interest

There are no conflicts to declare.

## Supplementary Material

RA-015-D5RA04860E-s001

## Data Availability

The authors confirm that the data supporting the findings of this study are available within the article and its SI. Supplementary information is available. See DOI: https://doi.org/10.1039/d5ra04860e.
