# Supplementary material for: Dual-luminescent Sc2(MoO4)3:Dy3+/Eu3+ phosphor system: energy transfer dynamics and high-sensitivity temperature sensing
Source: RSC Adv. 2025 Aug 15;15(35):28994–9002. doi: 10.1039/d5ra04860e (PMC12376941; doi:10.1039/d5ra04860e)
Supplement: RA-015-D5RA04860E-s001 [file RA-015-D5RA04860E-s001.pdf]

Supplementary Information (SI) for RSC Advances.
This journal is © The Royal Society of Chemistry 2025

Dual-Luminescent $Sc_2(MoO_4)_3$:Dy^{3+}/Eu^{3+} Phosphor System: Energy Transfer Dynamics and High-Sensitivity Temperature Sensing

Zhengrong Xia[a], Rongqing Li[a], Fangfang Liu[a], Weiwei Zhou[a,*], Wang Zhao[a], Mingjun Song[c,*] Junpeng Xue[b,*]

[a] Anhui Engineering Research Center for Photoelectrocatalytic Electrode Materials, School of Electrical Engineering, Huainan Normal University, Huainan 232038, China

[b] School of Science, Jiangsu University of Science and Technology, Zhenjiang 212100, China

[c] School of Chemistry, Chemical and Environmental Engineering, Weifang University, Weifang 261061, China

* Corresponding author.

E-mail address: wwzhou@hnnu.edu.cn (W.W. Zhou); smj521209@126.com (M.J. Song); xjplane@126.com (J. P. Xue)

1. Experimental section

1.1 Raw materials and synthesis

A series of SMO:$x$$Dy^{3+}$ (x = 0.01-0.08), SMO:0.02Eu^{3+}, and SMO:0.02Dy^{3+},$y$$Eu^{3+}$ (y = 0-0.09) phosphors were synthesized via a high-temperature solid-state reaction. Stoichiometric amounts of Sc_2O_3 (99.99%), MoO_3 (99.90%), Dy_2O_3 (99.99%), and Eu_2O_3 (99.99%) were precisely weighed, homogenized by mechanical mixing for 30 minutes. The two steps heating were adopted, firstly at 873 K for 6 h and then 1173 K for 12 h in atmospheric environment using muffle furnace. The heating and cooling rates employed in the synthesis process were both 3 K/min.

1.2 Characterization techniques

Phase purity was verified by XRD measurements using a Rigaku D/Max2000 diffractometer. The morphology and particle size of the phosphors were analyzed with a FEI Nova NanoSEM450 scanning electron microscope (SEM). UV-vis DRS were acquired using a Shimadzu UV3600 PLUS spectrophotometer. PL and PL excitation (PLE) spectra were recorded on a fluorescence spectrophotometer (model F-4600, Hitachi Ltd., Tokyo, Japan) equipped with a 150 W xenon arc lamp. All characterizations were performed at room temperature under ambient conditions.

1.3 DFT calculations and Rietveld refinement

All density functional theory (DFT) calculations were performed using the Vienna Ab initio Simulation Package (VASP).[1,2] The exchange-correlation functional was treated with the Perdew-Burke-Ernzerhof (PBE) generalized gradient approximation (GGA).[3] Electron-ion interactions were described using the projector augmented wave (PAW) method.[4] A plane-wave basis set cutoff energy of 400 eV was employed, and Brillouin zone integration was sampled using a 3×3×1 Monkhorst-Pack *k*-point mesh. Self-consistent field iterations were converged to thresholds of 10^{-4} eV for the total energy and 0.02 eV $Å^{-1}$ for atomic forces. Van der Waals interactions were incorporated using the DFT-D3 correction scheme.[5] Rietveld structural refinement of

$Sc_2(MoO_4)_3$: 0.02Dy^{3+}, 0.02Eu^{3+} phosphors was performed using the General Structure Analysis System-II (GSAS-II) software. The baseline and peak shape were fitted using Chebyshev1 and pseudo-Voigt functions, respectively, with the structure of $Sc_2(MoO_4)_3$ serving as the original model.

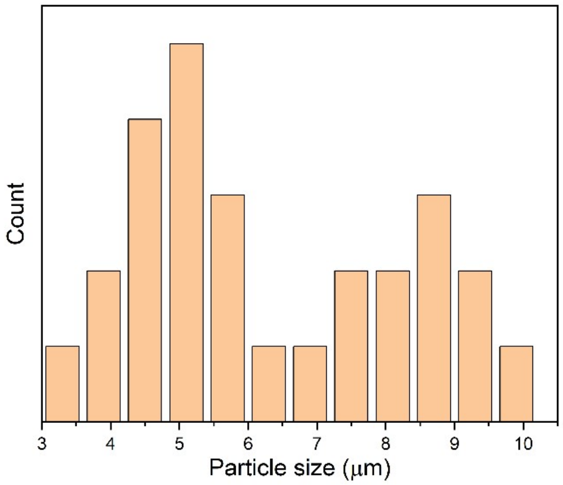

Figure S1 Particle size distribution of the $Sc_2(MoO_4)_3$: 0.02Dy^{3+}, 0.02Eu^{3+} phosphors.

References

1 G. Kresse and J. Hafner, *Phys. Rev. B*, 1993, **47**, 558-561.

2 G. Kresse and J. Hafner, *Phys. Rev. B Condens. Matter*, 1994, **49**, 14251–14269.

3 J. P. Perdew, K. Burke and M. Ernzerhof, *Phys. Rev. Lett.*, 1996, **77**, 3865-3868.

4 G. Kresse and D. Joubert, *Phys. Rev. B Condens. Matter*, 1999, **59**,1758–1775.

5 S. Grimme, S. Ehrlich and L. Goerigk, *J. Comput. Chem.*, 2011, **32**, 1456.
